# Coronavirus Antibodies in African Bat Species

**DOI:** 10.3201/eid1309.070342

**Published:** 2007-09

**Authors:** Marcel A. Müller, Janusz T. Paweska, Patricia A. Leman, Christian Drosten, Klaus Grywna, Alan Kemp, Leo Braack, Karen Sonnenberg, Matthias Niedrig, Robert Swanepoel

**Affiliations:** * Robert Koch-Institut, Berlin, Germany; † National Institute for Communicable Diseases, Sandringham, Republic of South Africa; ‡ Bernhard Nocht Institute for Tropical Medicine, Hamburg, Germany; § Conservation International, Cape Town, Republic of South Africa; ¶ EUROIMMUN AG, Lübeck, Germany

**Keywords:** group 4 coronavirus, SARS coronavirus, bat, SARS-like coronavirus, zoonosis, reservoir, emerging virus, serology, dispatch

## Abstract

Asian bats have been identified as potential reservoir hosts of coronaviruses associated with severe acute respiratory syndrome (SARS-CoV). We detected antibody reactive with SARS-CoV antigen in 47 (6.7%) of 705 bat serum specimens comprising 26 species collected in Africa; thus, African bats may harbor agents related to putative group 4 CoV.

Severe acute respiratory syndrome (SARS) emerged as a newly recognized human disease in the People’s Republic of China late in 2002 and spread globally, causing 8,422 infections with 916 (11%) deaths before it was brought under control in 2003 (*1*). The causative agent was identified as a coronavirus (SARS-CoV) (*2*–*4*), and related viruses found in palm civets (*Paguma larvata*), raccoon dogs (*Nycereutes procyonoides*) (*5*), and insectivorous bats in Asia cluster phylogenetically together with SARS-CoV in a putative group 4 (*6*–*10*). Farmed food animals such as civets may acquire SARS-like-CoV infection from bats, and adaptation of the viruses to these secondary hosts may occasionally give rise to strains capable of spreading and causing disease in humans (HCoV) (*10*).

## The Study

Bat serum specimens (n = 705) collected from 1986 through 1999 in South Africa (SA) and the Democratic Republic of the Congo (DRC) were tested. The first 248 serum specimens were collected from 1986 through 1989 in the Mpumalanga and Limpopo Provinces of SA for studies on rabies-related viruses, with the approval of the provincial Directorates of Nature Conservation and the Animal Ethics Committee of the University of the Witwatersrand. The remaining 457 serum samples were collected in 1995–1999 in the Bandundu and Oriental Provinces of the DRC for studies on Ebola and Marburg viruses, under the auspices of the International Committees for the Control of Ebola hemorrhagic fever in Kikwit, and Marburg hemorrhagic fever in Durba-Watsa, coordinated by the World Health Organization on behalf of the government of DRC. Bats were caught in mist nets, anesthetized, and exsanguinated by cardiac puncture. Serum specimens were stored at –70°C until analyzed.

For screening of serum specimens we used the SARS-CoV ELISA kit (EUROIMMUN AG, Lübeck, Germany) with minor modifications. Bat serum samples were tested at a dilution of 1:50, and horseradish peroxidase–labeled goat antibat immunoglobulin (Ig) conjugate (Bethyl, Montgomery, AL, USA) was used as secondary antibody at a dilution of 1:2,000. Negative bat serum was obtained from a captive-bred *Rousettus aegyptiacus* at the National Institute for Communicable Diseases, Sandringham, SA. The cut-off was determined as 3× the mean optical density value at 450/605 nm observed in negative control samples. Positive serum samples were retested and their titers determined. To evaluate test specificity and to exclude possible cross-reactivity to other viruses, especially to HCoVs, which have a seroprevalence in humans >90% (*11*), 662 human serum specimens were screened (Technical Appendix), including those from 90 patients with other acute respiratory infections, 70 HCoV-229E–positive serum specimens and 4 HCoV-NL63–positive serum specimens (provided by L. van der Hoek).

A confirmatory Western blot (WB) was done by using protein lysates from Vero E6 cell cultures (American Type Culture Collection [Manassas, VA, USA] CRL 1586) infected with SARS-CoV Hong Kong isolate 6109 (GenBank accession no. AY278491) and from uninfected Vero E6 cultures. Bat serum specimens were applied at dilutions of 1:500 and 1:2,000. Secondary detection was performed with the SuperSignal West Dura Extended Substrate chemiluminescence detection assay (Pierce Biotechnology, Rockford, IL, USA). The signal intensity of the 150-kDa spike (S), 50-kDa nucleocapsid (N) proteins was evaluated independently by 2 operators. For a second confirmatory WB, recombinant SARS-CoV proteins were used. For prokaryotic expression of recombinant SARS-CoV N protein and a fragment of the spike S protein (amino acid positions 318–510), we followed the instructions of the Champion pET Directional TOPO Expression kit (Invitrogen, Karlsruhe, Germany) using plasmids pET101-N and pET102-Saa318–510. Purification and refolding of the protein on column were done as described previously (*12*). Purified recombinant protein (15 μg) was resolved by electrophoresis on a discontinuous 12% sodium dodecyl sulfate–polyacrylamide gel. After blotting, nitrocellulose strips were incubated with bat serum samples diluted 1:2,500 and 1:5,000. One positive bat serum specimen was used as a reference to exclude variations in experimental procedures and signal intensities. Serum specimens that produced signals at a dilution 1:5,000 were considered positive as none of the control serum specimens showed reactivity at that dilution. To evaluate assay specificity, we tested 19 control serum specimens comprising 12 randomly selected bat serum specimens that were negative by ELISA, 2 SARS-CoV–positive human serum specimens, and 5 SARS-CoV–negative human serum specimens, including 4 HCoV-NL63–positive serum specimens.

A commercial indirect immunofluorescence test (SARS-CoV-IFTII kit, EUROIMMUN AG) was carried out as described by the manufacturer, except that bat serum samples were diluted 1:100, and slides were incubated at room temperature for 2 hours. Reactions were detected with goat-antibat immunogolublin (Ig) (Bethyl) at a dilution of 1:1,000 and fluorescein isothiocyanate–labeled donkey-antigoat Ig (Dianova, Hamburg, Germany) at a dilution of 1:100. Specificity of the indirect immunofluorescence test (IIFT) was determined by screening 572 human serum specimens. The sensitivity and correlation of IIFT versus ELISA were analyzed (Technical Appendix). In addition, the 19 selected control serum samples were tested.

Virus neutralization tests were performed as described elsewhere (*13*) except for using Vero E6 cells cultured in Dulbecco’s modified Eagle medium and SARS-CoV Hong Kong isolate 6109 (3.25 × 10^7^ PFU/mL, diluted 1:5,000). Bat serum dilutions in quadruplicate ranged from 1:10 to 1:320. After incubation at 37°C in 5% CO_2_ for 3 days, the cells were fixed with 8% formaldehyde and results interpreted as described (*13*).

Viral RNA was extracted from serum by using a QIAamp viral RNA extraction kit (QIAGEN, Hilden, Germany), and reverse transcription–PCR (RT-PCR) was performed essentially as described elsewhere (*14*), with the exception that 140 μL was not available from every bat. In such cases, input volume was reduced and replaced with water. A minimum of 20 μL was usually tested.

**Figure 1:**
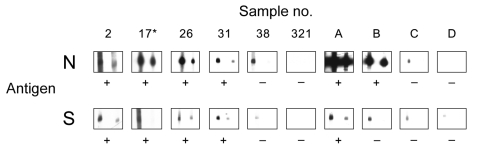
Results of Western blot analysis with recombinant severe acute respiratory syndrome–associated coronavirus (SARS-CoV) nucleocapsid (N) and spike (S) protein. Shown are examples for SARS-CoV ELISA–positive (2, 17, 26, 31) and –negative (38, 321) bat serum specimens tested using full-length recombinant SARS-CoV N and a fragment of the S protein (amino acids 318–510). Serum specimens were diluted 1:2,500 (left strips) and 1:5,000 (right strips). Secondary detection was performed by incubating the nitrocellulose strips with horseradish peroxidase (HRP)–labeled goat-antibat immunoglobulin (Ig) (Bethyl, Montgomery, AL, USA) (1:10,000). For chemiluminescence, SuperSignal Dura substrate (Pierce, Rockford, IL, USA) was added and films exposed for 1 min. Serum 17* was used as a reference for comparing blots. For evaluation purposes, strips were also incubated with human SARS-CoV–positive (A, B) and –negative serum specimens C and D (HCoV-NL63 positive) at the same dilutions, using goat-antihuman Ig HRP (1:20,000) for secondary detection. Serum specimens that produced signals at a dilution of 1:5,000 were recorded as positive (+).

**Figure 2:**
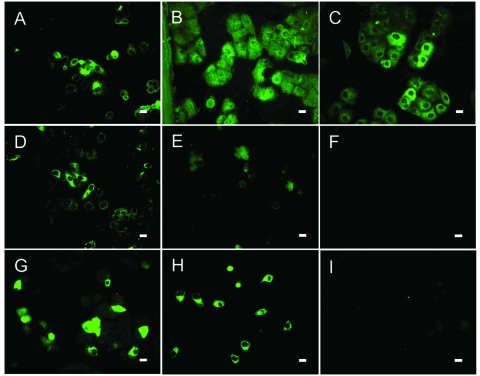
Results of indirect immunofluorescence (IF) test with Vero E6 cells infected with severe acute respiratory syndrome–associated coronavirus (SARS-CoV). The SARS-CoV diagnostic IIFT kit (EUROIMMUN AG, Lübeck, Germany) was used with minor modifications: bat and reference human serum specimens were diluted 1:100 (found to be the optimal dilution for bat sera) in sample buffer, and secondary detection was performed with goat-antibat immunoglobulin (Ig) (Bethyl, Montgomery, AL, USA) followed by fluorescein isothiocyanate (FITC)–labeled donkey-antigoat Ig (Dianova, Hamburg, Germany) (A–F) or FITC-labeled goat-antihuman Ig (G–I). Frames A–D, SARS-CoV ELISA–positive bat serum specimens 2, 17, 26, 31; E–F, ELISA-negative bat serum specimens 38 (showing unspecific signals) and 306; G–H, SARS-CoV–positive human control serum specimens A and B; I, negative human serum C. All photographs were taken at equivalent microscope settings. Scale bars represent 20 μm.

Antibody activity to SARS-CoV antigen was detected by ELISA in 7 of 26 bat species tested at both collection sites with a seroprevalence of 6.7% (47/705). The highest prevalences were found in the fruit bat *Rousettus aegyptiacus* (Chiroptera: Pteropodidae) (16.4%) and the insectivorous bat *Mops condylurus* (Chiroptera: Molossidae) (12.2%) (Table). ELISA titers ranged from 50 (73% of the serum samples) to 800. Confirmatory WB analyses performed by 2 methods on ELISA-positive samples for which sufficient material remained available, were positive in 36 (97.3%) of 37 serum specimens, but IIFT was positive in only 12 (32.4%) of 37 samples (Table; Figures 1, 2; Figure in Technical Appendix). None of the assays used detected antibodies to other human pathogenic coronaviruses (Technical Appendix; Figures 1, 2). Neutralizing activity to SARS-CoV was not found in any of the ELISA-positive samples, and RT-PCR did not detect CoV nucleic acid in 262 serum specimens tested (data not shown).

**Table:** Antibody to SARS-CoV in bat sera collected in 1986–1999 at 4 locations in central and southern Africa*

	ELISA: positive/tested (%)†					WB: positive/ tested‡	IIFT: positive/ tested‡
	Limpopo Province, SA	Mpumalanga Province, SA	Oriental Province, DRC	Bandundu Province, DRC	Total		
Fruit bats							
*Casinycteris argynnis*				0/3	0/3		
*Eidolon helvum*				0/6	0/6		
*Epomophorus gambianus*	0/4	0/6			0/10		
*Epomophorus wahlbergi*	0/2				0/2		
*Epomops franqueti*				0/5	0/5		
*Hypsignathus monstrosus*				1/11 (9.1)	1/11 (9.1)	1/1	0/1
*Lyssonycteris angolensis*			1/16 (6.3)	0/2	1/18 (5.6)	1/1	0/1
*Myonycteris torquata*				1/7 (14.3)	1/7 (14.3)		
*Rousettus aegyptiacus*	11/29 (37.9)		17/142 (12.0)		28/171 (16.4)	26/26	7/26
Insect bats							
*Chaerephon pumila*	0/35	0/18		0/1	0/54		
*Hipposideros caffer*	0/5		0/9		0/15		
*Hipposideros commersoni*			0/16		0/16		
*Miniopterus inflatus*			1/34 (2.9)		1/34 (2.9)		
*Miniopterus schreibersi*	0/1				0/1		
*Mops condylurus*	3/19 (15.8)	11/96 (11.5)			14/115 (12.2)	8/9	5/9
*Mops midas*	0/15				0/15		
*Myotis bocagei*	0/1				0/1		
*Nycteris argae*			0/1		0/1		
*N. thebaica*	0/6				0/6		
*Pipistrellus capensis*	0/1				0/1		
*Rhinolophus darlingi*	0/1				0/1		
*Rhinolophus landeri*	0/2				0/2		
*Rhinolophus fumigatus*			1/204 (0.5)		1/204 (0.5)		
*Scotophilus borbonicus*	0/1				0/1		
*S. dinganii*	0/5				0/5		
*Taphozous mauritianus*	0/1				0/1		
Totals	14/128 (10.9)	11/120 (9.2)	20/422 (4.7)	2/35 (5.7)	47/705 (6.7)	36/37	12/37

*SARS-CoV, severe acute respiratory syndrome–associated coronavirus; SA, South Africa; DRC, Democratic Republic of Congo; WB, Western blot; IIFT, indirect immunofluorescence test. †Serum specimens were screened for antibody by modification of a commercially available ELISA kit. Titers ranged from 1:50 to 1:800. ‡Confirmatory tests were performed by 2 WB analyses and IIFT when sufficient sample was available.

## Conclusions

The results of WB analyses support the specificity of the ELISA used in this study. The IF test is known to be less sensitive than ELISA but still provided confirmation in one third of the serum specimens tested. The negative results in the viral neutralization tests are not unexpected because this assay detects only antibodies that interfere with the specific entry mechanism of SARS-CoV, and putative group 4 CoVs from African bats may not use it. Moreover, deletions and mutations found in Asian bat SARS-like–CoV isolates lie in the S protein region essential for binding of SARS-CoV to the cellular receptor, angiotensin-converting enzyme 2, and thus are likely to affect cross-neutralization, as emphasized by conflicting results obtained in Asia (*6*,*7*,*15*). The negative findings obtained in RT-PCR can be explained by the unlikelihood of finding virus nucleic acid in serum. Studies in Asia used rectal swabs instead of serum samples, and the virus likely persists in the enteric tract but may not be found in serum at all.

Both bat species (*R. aegyptiacus* and *M. condylurus*) are widely distributed in Africa but vary in the degree of contact with humans. *R. aegyptiacus* roosts in caves but forages in orchards, whereas *M. condylurus* roosts in buildings. The results of this preliminary study suggest that some of the African bat species harbor agents related to putative group 4 CoV, and therefore further investigations should be undertaken to determine potential public health risks.

## Supplementary Material

Technical Appendix Evaluation of EUROIMMUN Anti-SARS-CoV IIFT and ELISA*
