# Supplementary material for: Coronavirus Antibodies in African Bat Species
Source: Emerg Infect Dis. 2007 Sep;13(9):1367–70. doi: 10.3201/eid1309.070342 (PMC2857293; doi:10.3201/eid1309.070342)
Supplement: Technical Appendix — Evaluation of EUROIMMUN Anti-SARS-CoV IIFT and ELISA* [file 07-0342_Techapp-s1.pdf]

Technical Appendix.

Evaluation of EUROIMMUN Anti-SARS-CoV IIFT and ELISA*

Specificity

Serum panel	n (IIFT)	SARS-CoV IIFT (IgG)	n (ELISA)	SARS-CoV ELISA (IgG)
SARS contact persons	118	0	ND	0
HIV, HCV, or HBV patients	90	0	ND	0
Patients with other acute respiratory tract infections	90	0	90	0
HCoV-229E positive	70	0	70	0
HCoV-NL63 positive	4	0	4	0
Blood donors (Germany)	200	0	401	1
Blood donors (China)	ND	ND	97	0
Total	572	0	662	1

SARS-CoV IIFT specificity (IgG): 100%.

SARS-CoV ELISA specificity (IgG): >99%.

Sensitivity

Serum panel	n	SARS-CoV IIFT–positive IgG
SARS patients†, Germany	9	9 (100%)
SARS patients†, People’s Republic of China	147	144 (98%)

SARS-CoV IIFT sensitivity (IgG): 98%–100%.

SARS-CoV IIFT versus SARS-CoV ELISA (n = 34‡)

		SARS-CoV IIFT	
		Positive	Negative
SARS-CoV ELISA	Positive	23	0
	Negative	0	11

Correlation: 100%.

*SARS-CoV, severe acute respiratory syndrome–associated coronavirus; IIFT, indirect immunofluorescence test; Ig, immunoglobulin; ND, not done; HCV, hepatitis C virus; HBV, hepatitis B virus.

†>10 d after onset of symptoms.

‡34 serum specimens from SARS patients (You An Hospital, Beijing, People’s Republic of China); samples were taken 1–55 d after onset of symptoms.

Technical Appendix Figure. Western blot (WB) analysis with protein lysates of severe acute respiratory syndrome–associated coronavirus (SARS-CoV)–infected and control Vero E6 cells. ELISA-positive bat serum specimens were tested in WB analysis with cell lysates of SARS-CoV–infected Vero E6 cells (+) and noninfected cell lysates as a control for nonspecific reactions to cellular proteins (–). Sera were diluted 1:500 and nitrocellulose strips incubated at room temperature for 2 h. Secondary detection was performed as in the recombinant WB, but films were exposed for 10 s. Results obtained with positive bat serum 26 (lanes 1, 2) and negative bat serum 38 (lanes 3, 4) are illustrated. Lane 1 shows characteristic bands at the 50-kDa nucleocapsid (N) and 150-kDa spike (S) protein positions.
